# A Case Report and Literature Review of Nonketotic Hyperglycemic Hemichorea

**DOI:** 10.7759/cureus.56087

**Published:** 2024-03-13

**Authors:** Lisle W Blackbourn, Manjari Uppu, Zeeshan Zubair, Deepak Nair

**Affiliations:** 1 Neurology, University of Illinois COM (College of Medicine) Peoria, Peoria, USA; 2 Neurology, OSF Illinois Neurological Institute, Peoria, USA; 3 Medicine, University of Illinois COM (College of Medicine) Peoria, Peoria, USA

**Keywords:** basal ganglia dysfunction, choreiform movements, diabetes, diabetic striatopathy, nonketogenic hyperglycemic hemichorea

## Abstract

Nonketogenic hyperglycemic hemichorea, also recognized as diabetic striatopathy, is a rare manifestation of diabetes mellitus. The diagnosis of nonketotic hyperglycemic hemichorea is usually made through imaging along with a corresponding clinical picture. Early identification and treatment can lead to complete resolution of the symptoms and better patient outcomes. Here we present a 49-year-old female patient, with a past medical history of poorly controlled type 2 diabetes mellitus and prior left index finger amputation as a complication of her diabetes, who presented for evaluation of a two-week duration of sudden-onset left upper extremity choreiform movements.

## Introduction

Nonketogenic hyperglycemic hemichorea, also recognized as diabetic striatopathy, is a rare manifestation of diabetes mellitus. Often presenting as a distinct neurological manifestation, it is characterized by choreiform movements and basal ganglia dysfunction [1]. While diabetes is widely recognized for its vascular and metabolic complications, the involvement of the central nervous system in the form of diabetic striatopathy remains a less-explored facet. This condition, often associated with poorly controlled diabetes, manifests as involuntary movements and neuroimaging abnormalities within the striatum [2]. The estimated prevalence is believed to be 1 in 100,000 worldwide [2]. Understanding the clinical presentation, diagnostic challenges, and management strategies of diabetic striatopathy is essential for clinicians navigating the complex landscape of diabetes-related complications. There is a long list of differentials that can cause such movements including infectious, demyelinating, stroke, vasculitis, paraneoplastic, or metabolic causes [3]. This can make it a diagnostic challenge and cause one to overlook having the diagnosis be a diabetic complication. Thus, understanding the overall clinical picture is important to correctly diagnose the patient. Strict glycemic control is the main treatment of diabetic striatopathy to reduce symptoms in the long term, however, various medications can be used for symptomatic treatment in the short term [2]. In this context, we present a case study of a 49-year-old female patient, shedding light on the intricacies of diabetic striatopathy and its implications for both clinical practice and the broader understanding of diabetes-associated neurological manifestations.

## Case presentation

A 49-year-old female patient with a past medical history of poorly controlled type 2 diabetes mellitus (T2DM) and prior left index finger amputation as a complication from her diabetes presented for evaluation of a two-week duration of sudden onset left upper extremity choreiform movements, which were progressing as more frequent and intense in nature. The patient had completely retained awareness and alertness during these events and was unable to suppress movements voluntarily.

The patient was initially diagnosed with T2DM about 20 years ago with a family history significant for type 1 diabetes mellitus in her maternal aunt, grandmother, and great-grandmother. She initially was prescribed metformin for one year and then began managing with diet and exercise due to personal preferences. She was admitted for necrotizing fasciitis in 2019 with a reported hemoglobin A1c (HbA1c) to be 15% at that time. The patient was subsequently placed on insulin. She continued insulin until 2021 and her HbA1c was reported to be around 7-8%. The patient then again began managing with diet and exercise due to personal preferences despite medical recommendations to continue insulin. Since then, the patient says she was not seen by a primary care doctor, and her last HbA1c was drawn two years prior.

On exam, the patient had frequent choreiform movements with flailing of the left upper extremity. The muscle testing, tone, and reflexes that were able to be gathered were normal. The patient had a sensory loss in the bilateral lower extremities in a stocking distribution.

**Figure 1:**
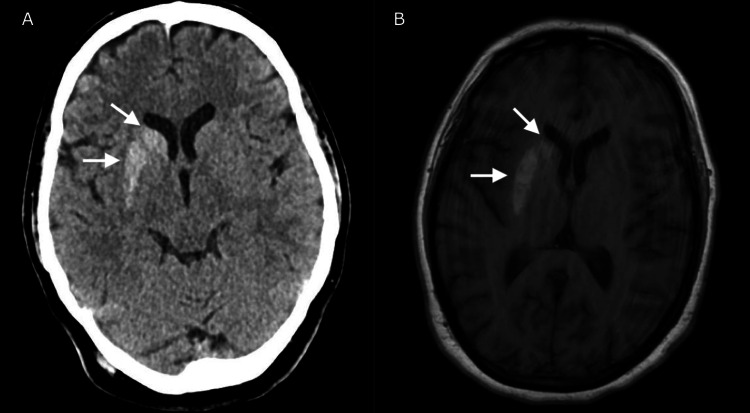
CT head (A) and MRI brain (B) showing a hyperdense and hyperintense lesion respectively in the right basal ganglia.

The patient had normal vitals, including no fever. Initial laboratory blood work, including a complete blood count and complete metabolic panel, was unremarkable outside of an elevated blood glucose level of 184 mg/dL. A noncontrast CT head on arrival to the emergency department showed a hyperdense lesion. At this point, there was concern for a hemorrhage versus a mass lesion, and neurology was then consulted. An MRI brain without contrast showed a hyperintense lesion in the right basal ganglia on T1 imaging with no strong restricted diffusion or fluid-attenuated inversion recovery (FLAIR) correlate thus indicating no hemorrhage, stroke, or mass was present. Imaging findings can be seen in Figure 1.

HbA1c later resulted as >14%. Anti-GAD and anti-IA 2 antibodies were negative.

Given the clinical picture and imaging findings, a diagnosis of nonketogenic hyperglycemic hemichorea was made. The patient was trialed on a number of medications, including olanzapine and benztropine before clonazepam was used to help manage the choreiform movements in the short term. Strict diabetic control was the goal for the patient to achieve better control of the movements in the long term. The patient was restarted on an insulin regimen while in the hospital with the help of internal medicine and eventually was discharged on 7 units of lispro three times a day with meals and 20 units of glargine nightly. The patient was also set up with a primary care doctor to follow up with as an outpatient.

## Discussion

The presented case of a 49-year-old female with poorly controlled type 2 diabetes mellitus and sudden-onset left upper extremity choreiform movements highlights the intricate relationship between diabetes and central nervous system manifestations, particularly diabetic striatopathy. The distinct choreiform movements observed in our patient are indicative of basal ganglia dysfunction, a phenomenon increasingly recognized in the spectrum of diabetes-related neurologic complications [4]. Imaging findings corresponding to the unilateral hyperdensity and hyperattenuation in the right basal ganglia confirmed the diagnosis and clinical picture of this patient as most compatible with nonketotic hyperglycemic hemichorea [2]. The patient’s elevated HbA1c (>14%) on admission further highlights the association between poor glycemic control and the development of diabetic striatopathy.

While the overall pathogenic mechanism of nonketotic hyperglycemic hemichorea is unclear, as well as the cause of the neuroradiological findings, the hypothesized mechanisms include hyperviscosity from hyperglycemia leading to blood-brain barrier disruption and decreased GABA in the striatum due to the nonketotic state [5]. In the management of choreiform movements, a trial of various medications preceded the successful short-term control achieved with clonazepam. However, the long-term focus shifted toward strict diabetic control, recognizing the potential for sustained improvement in neurological symptoms with glycemic control. Treatment includes aggressive glucose control, which can lead to both the resolution of the hemichorea as well as the imaging findings [4].

A literature search was completed on PubMed of “diabetic striatopathy” and can be seen in Table 1, with 37 cases being found in our search [2,6-33].

**Table 1:** Previously published cases of diabetic striatopathy

Year of Report	Age	Gender	Treatment	Patient Outcome	Reference Number
2013	73	Female	Haloperidol and glycemic control	Bulbar weakness had improved and generalized chorea resolved	[6]
2016	79	Male	Glycemic control	Symptoms completely resolved and decreased signal intensity in both the basal ganglia	[7]
2017	57	Female	Olanzapine and clonazepam	Lost to follow-up	[8]
2018	64	Male	Haloperidol and glycemic control	Symptom improvement and movements disappeared during his sleep	[9]
2019	69	Female	Valproic acid and haloperidol	Relapse episode of hemichorea-hemiballism then full recovery	[10]
2019	53	Male	Glycemic control	Not reported	[11]
2020	90	Male	Tiapride and glycemic control	Abnormal movements resolved but striatal hyperdensity was still present at the follow-up CT scan after 10 days but was less pronounced	[12]
2020	12	Female	Haloperidol, tetrabenazine, clonazepam, valproate, and glycemic control	Minimal symptoms after 45 days	[13]
2020	20	Female	Clonazepam, tetrabenazine, and glycemic control	Symptoms resolved completely	[14]
2021	70	Male	Glycemic control	Improvement of symptoms and almost complete resolution six months later on MRI	[15]
2021	18	Male	Glycemic control	Lost to follow-up	[16]
2021	88	Female	Glycemic control	Passed away from other complications during the hospital stay	[17]
2021	71	Female	Glycemic control	Symptoms resolved completely	[18]
2021	67	Male	Glycemic control	Symptoms resolved completely and there was a persistence of striatal abnormalities in neuroimaging at six months	[19]
2021	61	Female	Glycemic control	Symptoms resolved completely and persistence of striatal abnormalities in neuroimaging at 6 months	[19]
2022	89	Female	Haloperidol and glycemic control	Lost to follow up	[20]
2022	86	Female	Clonazepam and glycemic control	Lost to follow up	[21]
2022	86	Male	Haloperidol and glycemic control	Three-month follow-up showed complete resolution of involuntary movements and imaging findings	[22]
2022	70	Female	Tetrabenazine	Symptom persistence at three months	[23]
2022	72	Female	Glycemic control and symptomatic treatment	Symptoms resolved completely	[24]
2022	74	Female	Glycemic control	Symptoms improved	[24]
2022	76	Female	Diazepam, phenobarbital, sodium valproate, and glycemic control	Symptoms improved	[24]
2022	80	Male	Phenytoin and glycemic control	Symptoms resolved completely	[24]
2022	90	Female	Glycemic control	Symptoms improved	[24]
2022	73	Female	Glycemic control	Symptoms improved and repeat CT showed decreased density and range of lesions in the left basal ganglia	[24]
2022	68	Male	Trihexyphenidyl, clonazepam, and glycemic control	Symptoms resolved completely and complete resolution of imaging findings at 18 months	[25]
2022	51	Male	Tetrabenazine and glycemic control	Symptoms improved	[26]
2022	55	Male	Glycemic control	Symptoms resolved at discharge but lost to follow-up	[27]
2022	45	Male	Haloperidol and glycemic control	Symptoms improved	[28]
2022	11	Female	Trihexyphenidyl and glycemic control	Lost to follow-up	[29]
2022	10	Female	Trihexyphenidyl, clonazepam haloperidol, and glycemic control	Symptoms improved	[29]
2022	75	Female	Levetiracetam, tetrabenazine, and glycemic control	Symptoms improved	[30]
2023	73	Male	Glycemic control	Symptoms improved	[31]
2023	83	Female	Tetrabenazine	Symptoms resolved completely at 3 months but lost to follow-up thereafter	[32]
2023	72	Male	Glycemic control	Symptoms resolved completely	[2]
2023	Early 70s	Male	Haloperidol, risperidone, and glycemic control	Symptoms resolved completely and MRI findings resolved at 13 weeks	[33]

Most cases showed substantial improvement or complete resolution of symptoms with proper glycemic control. Treatment for symptomatic movement control varied and was based on what worked best for the patient while in the hospital. Some cases even reported seizure activity coming from the lesions seen in brain imaging. Our search also revealed that this complication was not limited to adults and could arise in the pediatric population.

Seven of the 37 cases found were lost to follow-up. This can be a common challenge in chronic medical conditions, and these patients usually face poorer disease control. This would seem unsurprising given the need for poorly controlled diabetes to cause this complication but highlights the need for close follow-up to make sure strict diabetic control can be achieved in order to have symptom resolution. Those cases that did follow-up imaging showed a resolution of the imaging findings, which varied from 3 months to 18 months in time.

## Conclusions

In conclusion, this case demonstrates a unique relationship between poorly controlled diabetes, basal ganglia dysfunction, and choreiform movements. The diagnosis of nonketotic hyperglycemic hemichorea is usually made through imaging along with a corresponding clinical picture. Early identification and treatment can lead to complete resolution of the symptoms and better patient outcomes. Clinicians should remain vigilant for such presentations in diabetic patients, fostering a comprehensive approach that encompasses both symptomatic relief and glycemic control for optimal patient outcomes.

## References

[1] Collado-Saenz J, Baeza-Trinidad R (2022). Nonketotic hyperglycemic hemichorea. N Engl J Med.

[2] Chithrapathra KE, Hewanayake WS, Egodage S, Silva S (2023). Diabetic striatopathy: a case report of a patient with poor glycaemic control and abnormal movements. Cureus.

[3] Cardoso F, Seppi K, Mair KJ, Wenning GK, Poewe W (2006). Seminar on choreas. Lancet Neurol.

[4] Lam PL, Iu PP, Cho DH (2023). Non-ketotic hyperglycaemic hemichorea: a rare complication of uncontrolled diabetes mellitus. Hong Kong Med J.

[5] Lau SC, Tan SM (2023). Nonketotic hyperglycemic hemichorea. Am J Med.

[6] Singh A (2013). Diabetic striatopathy. Mayo Clin Proc.

[7] Udare AS, Sankhe S, Mondel PK (2016). Bilateral diabetic striatopathy. Asian J Neurosurg.

[8] Lucassen EB, Delfyett WT, Stahl MC (2017). Persistent hemichorea and caudate atrophy in untreated diabetic striatopathy: a case report. Case Rep Neurol.

[9] Vasudevan V, Laway BA, Wani AI, Wani MM (2018). Chorea associated with nonketotic hyperglycemia (diabetic striatopathy) in an elderly male. Indian J Endocrinol Metab.

[10] Lin YT, Chen SC, Yip PK, Wang V (2019). Magnetic resonance imaging volumetric analysis for diabetic striatopathy with two episodes of hemichorea-hemiballism syndrome: a case report. Medicine (Baltimore).

[11] Chua CB, Chen HC, Su HY, Tsai IT, Sun CK (2019). Images of the month 1: diabetic striatopathy without hemichorea/hemiballism. Clin Med (Lond).

[12] Mikulenka P, Stetkarova I (2020). Hemichorea in ketotic hyperglycemia with hyperdense striatum mimicking hemorrhagic transformation in a patient using apixaban. Neuro Endocrinol Lett.

[13] Puneeth HR, Kulhalli P, Ratageri VH (2020). Diabetic striatopathy in a child: a cause of reversible chorea. Indian Pediatr.

[14] Lin JB, Sng AA, Wang FS, Tan AP, Han VX (2020). Acute hemichorea in a young type 1 diabetic. Int J Neurosci.

[15] Iri T, Yano H, Kinjo M (2021). Diabetic striatopathy. BMJ Case Rep.

[16] Tencer J, Yum SW (2021). Teaching neuroimage: basal ganglia T1 hyperintensity and SWI signal diabetic striatopathy in an 18-year-old man with type 1 diabetes mellitus. Neurology.

[17] Dong M, E JY, Zhang L, Teng W, Tian L (2021). Non-ketotic hyperglycemia chorea-ballismus and intracerebral hemorrhage: a case report and literature review. Front Neurosci.

[18] Homaida M, Kanodia AK, Young N, Yu WM (2021). Diabetic striatopathy: a rare condition and diagnostic dilemma. BMJ Case Rep.

[19] Markowska K, Koziorowska-Gawron E, Papier P, Koszewicz M, Budrewicz S, Bladowska J, Zimny A (2021). Easily missed or misinterpreted: diabetic striatopathy in the course of ketotic hyperglycaemia. Postgrad Med J.

[20] Ando Y, Kadoya M, Kodera T (2023). Involuntary movements during treatment for hyperglycemia. AACE Clin Case Rep.

[21] Mozzini C, Ghirardi R, Pagani M (2022). Diabetic striatopathy: case report and possible new actors. Case Rep Neurol Med.

[22] Godani M, Lanza G (2022). Diabetic striatopathy: parenchymal transcranial sonography as a supplement to diagnosis at the emergency department. Diagnostics (Basel).

[23] Lim KX, Khaing Zin T, Yu Z, Peh WM (2022). Delayed presentation of hemichorea in diabetic striatopathy. Cureus.

[24] Xu Y, Shi Q, Yue Y, Yan C (2022). Clinical and imaging features of diabetic striatopathy: report of 6 cases and literature review. Neurol Sci.

[25] Huang X, Qi J, Li Y, Li J, Yang MG (2022). Diabetic striatopathy complicated with acute ischemic stroke: a case report. Front Neurosci.

[26] Park G, Kesserwani HN (2022). A case report of diabetic striatopathy: an approach to diagnosis based on clinical and radiological findings. Cureus.

[27] Evers Smith CM, Chaurasia KK, Dekoski DC (2022). Non-ketotic hyperglycemic hemichorea-hemiballismus: a case of a male with diabetes mellitus and speech disturbances. Cureus.

[28] Safan AS, Sharma O, Almasri M, D'Souza AI, Suliman O (2022). Is diabetic striatopathy the culprit of seizures in a patient with ketotic hyperglycemia-induced hemichorea-hemiballismus?. BMC Neurol.

[29] Rai S, Kaul V, Singh S, Kaur S, Thenmurugan P (2022). Diabetic striatopathy: a new challenge in type 1 pediatric diabetic patients. Oman Med J.

[30] Chatterjee S, Ghosh R, Ojha UK, Diksha Diksha, Biswas P, Benito-León J, Dubey S (2022). Recurrent facial focal seizures with chronic striatopathy and caudate atrophy—a double whammy in an elderly woman with diabetes mellitus. Neurohospitalist.

[31] Li H, Cheng Y, Tang W, Hu Y, Jia G, Wu T, Wang K (2023). Cognitive decline as the main manifestation of diabetic striatal disease but without involuntary movements: a case report. BMC Neurol.

[32] Sperotto R, Ceccarelli L, Tereshko Y, Merlino G, Gigli GL, Valente M (2023). The possible precipitating role of SARS-CoV-2 in a case of late-onset hemichorea due to a hyperosmolar hyperglycemic state: case report and brief literature review. Medicina (Kaunas).

[33] Sawamura T, Karashima S, Kawahara H, Yoneda T (2023). Abnormal findings in the basal ganglia: a diagnostic clue for patients with diabetic striatopathy. BMJ Case Rep.

